# Chronic Palmitate Exposure Inhibits Insulin Secretion by Dissociation of Ca^2+^ Channels from Secretory Granules

**DOI:** 10.1016/j.cmet.2009.09.011

**Published:** 2009-12-02

**Authors:** Michael B. Hoppa, Stephan Collins, Reshma Ramracheya, Leanne Hodson, Stefan Amisten, Quan Zhang, Paul Johnson, Frances M. Ashcroft, Patrik Rorsman

**Affiliations:** 1 The Oxford Centre for Diabetes, Endocrinology, and Metabolism, Churchill Hospital, Oxford OX3 7LJ, UK; 2 Nuffield Department of Surgery, John Radcliffe Hospital, Oxford OX3 9DU, UK; 3 Department of Physiology, Anatomy, and Genetics, Oxford University, Oxford OX1 3PT, UK

**Keywords:** HUMDISEASE

## Abstract

Long-term (72 hr) exposure of pancreatic islets to palmitate inhibited glucose-induced insulin secretion by >50% with first- and second-phase secretion being equally suppressed. This inhibition correlated with the selective impairment of exocytosis evoked by brief (action potential-like) depolarizations, whereas that evoked by long (∼250 ms) stimuli was unaffected. Under normal conditions, Ca^2+^ influx elicited by brief membrane depolarizations increases [Ca^2+^]_i_ to high levels within discrete microdomains and triggers the exocytosis of closely associated insulin granules. We found that these domains of localized Ca^2+^ entry become dispersed by long-term (72 hr), but not by acute (2 hr), exposure to palmitate. Importantly, the release competence of the granules was not affected by palmitate. Thus, the location rather than the magnitude of the Ca^2+^ increase determines its capacity to evoke exocytosis. In both mouse and human islets, the palmitate-induced secretion defect was reversed when the β cell action potential was pharmacologically prolonged.

## Introduction

Type 2 diabetes mellitus is characterized by insufficient insulin secretion in response to elevations of plasma glucose (Weyer et al., 1999). This decreased insulin response shows a strong correlation with obesity (Kahn et al., 2006) and elevated plasma levels of free fatty acids (FFAs) (Boden, 2002). Protracted exposure to FFAs results in marked suppression of glucose-induced insulin secretion both in vivo and in vitro (Sako and Grill, 1990; Zhou and Grill, 1994). Although the latter effect has been extensively characterized, the cellular mechanisms remain enigmatic (Poitout and Robertson, 2008). The adverse effects of FFAs on β cell function, often referred to as “lipotoxicity” (Lee et al., 1994), have been postulated to culminate in β cell death. Indeed, experiments on human cells in vitro demonstrated marked apoptosis after just 24 hr exposure to elevated FFAs and glucose (El-Assaad et al., 2003). Apoptosis of β cells has been attributed to both oxidative stress (Morgan et al., 2007) and ER stress (Kharroubi et al., 2004) as well as other pathways (Cnop et al., 2005). However, other studies have failed to find evidence of any significant apoptosis following long-term exposure to FFAs (Kelpe et al., 2003). These discrepancies have been attributed to differences in the levels of unbound FFAs or islet viability (Poitout and Robertson, 2008).

In healthy β cells, elevation of glucose produces (via its metabolism) an increased cytoplasmic ATP/ADP ratio and closure of ATP-regulated K^+^ channels (K_ATP_ channels). This causes membrane depolarization and the opening of voltage-dependent Ca^2+^ channels. The resultant increase in [Ca^2+^]_i_ triggers the exocytosis of insulin-containing granules. This secretory response consists of a rapid but transient first phase followed a slowly developing second phase (Henquin et al., 2002). Exocytosis during brief (action potential-like) depolarizations primarily involves granules belonging to a readily releasable pool (RRP) in close proximity to the voltage-gated Ca^2+^ channels (Barg et al., 2001). In a previous study (Olofsson et al., 2007), we reported that inhibition of glucose-stimulated insulin secretion by long-term exposure to the FFAs oleate and palmitate occurred without any signs of increased β cell death, reduced insulin synthesis, impaired glucose metabolism, K_ATP_ channel regulation, or Ca^2+^ signaling. It was therefore proposed that the perturbation occurs at the level of exocytosis. Here we have examined this possibility in greater detail by measurements of lipid composition, membrane fluidity, imaging of submembrane [Ca^2+^]_i_, and exocytosis.

## Results

### Effects of Palmitate on Islet mRNA Content

The effects of long-term palmitate treatment on the amounts of mRNA encoding key proteins involved in granule priming and exocytosis were evaluated by quantitative PCR (qPCR; see Table S1 available online). No major changes in gene transcription were observed that would account for the strong inhibition of insulin secretion observed (Olofsson et al., 2007).

### Analysis of Palmitate Processing and Storage by Pancreatic Islets

**Figure 1:**
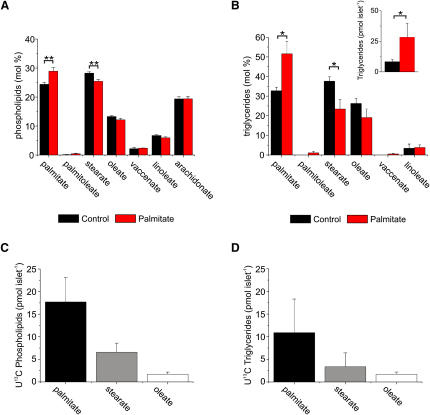
Pancreatic Islet Lipid Analysis (A) Proportion of individual phospholipid fatty acids in control (black) and palmitate-treated (red) islets. (B) Proportion of individual triglyceride fatty acids in control (black) and palmitate-treated (red) islets. The inset shows total TGs in islets exposed, or not, to palmitate. (C and D) Amounts of uniformly labeled ^13^C phospholipids (C) and triglycerides (D) per islet during 72 hr treatment with exogenous ^13^C-labeled palmitate. All phospholids and triglycerides labeled with ^13^C originate from ^13^C-labeled exogenous palmitate. All experiments were performed four times with a minimum of 220 islets per group. For each experiment, islets isolated from six mice were pooled and divided evenly into the two pools that were cultured for 72 hr in the absence or presence of palmitate. All error bars are ±SEM, ^∗^p < 0.05; ^∗∗^p *<* 0.01.

Changes in the lipid composition have been shown to affect the energy requirements of membrane fusion (Chernomordik and Kozlov, 2008; Furber et al., 2009). We therefore compared the phospholipid (PL) and triglyceride (TG) composition of palmitate-treated and control islets. The palmitate content of the phospholipid fraction increased by ∼3–4 mol%, and stearate content correspondingly decreased in islet exposed to palmitate (see definition in the Experimental Procedures) (Figure 1A). These changes are much larger than those observed in erythrocytes from animals fed a high-fat diet (Poppitt et al., 2005). The overall ratio of saturated to unsaturated fatty acids in the phospholipid fraction remained unaltered when comparing control and palmitate-treated groups (0.88 ± 0.03 and 0.81 ± 0.03; p < 0.13; n = 4). Exposure of islets to palmitate resulted in a >3-fold increase in overall TG levels (Figure 1B, inset). Whereas the palmitate content of the triglyceride fraction increased by ∼50%, the stearate content was reduced by one third. These findings are in good agreement with a previous study that measured total changes in fatty acid composition in MIN6 clonal cells (Busch et al., 2005).

We also made use of uniformly labeled (U^13^C) palmitate (Hodson et al., 2009) to track the incorporation of exogenous palmitate into phospholipids and triglycerides. In islets exposed to exogenous ^13^C-labeled palmitate for 72 hr, a significant amount of ^13^C-labeled palmitate was converted into phospholipids (Figure 1C), as well as some expected deposition as TGs (Figure 1D). Thus, palmitate is not simply deposited inside the β cell but is actively processed (by both desaturation and elongation) and is converted into phospholipids, the major component of the plasma membrane. No incorporation of palmitate into phospholipid was observed during a 2 hr incubation (data not shown).

### Membrane Fluidity Is Not Affected by Palmitate Treatment

Impaired insulin exocytosis may be secondary to changes in membrane fluidity resulting from the altered phospholipid composition. We therefore examined membrane fluidity by analyzing lipid mobility by fluorescence recovery after photobleaching (FRAP). Pancreatic β cells isolated from islets incubated for 72 hr in the presence or absence of palmitate were labeled with the 14-carbon lipids fluorescently tagged with individual BODIPY fluorophores. No palmitate-induced changes in membrane fluidity were detected by this method (Figure S1).

### Loss of Localized Ca^2+^ Influx Following Palmitate Treatment

**Figure 2:**
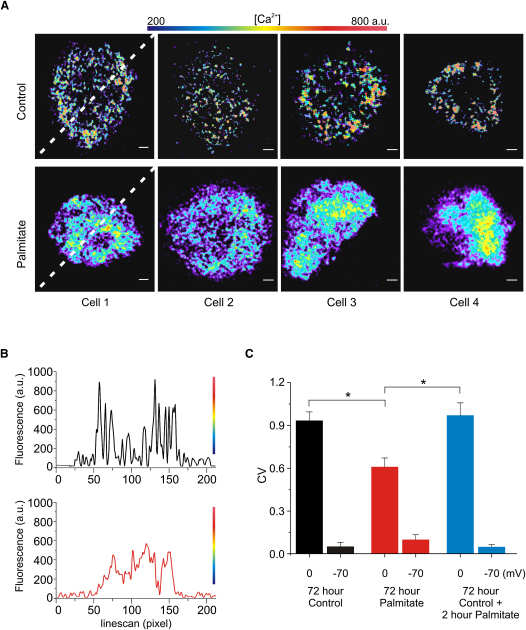
TIRF Imaging Reveals a Redistribution of Ca^2+^ Influx Sites in Cells after Palmitate Treatment (A) Evanescent field illumination of voltage-clamped cells infused with EGTA (10 mM) stimulated by a single 50 ms depolarization from −70 to 0 mV. Changes in [Ca^2+^]_i_ are displayed in pseudocolors with black/blue and yellow/red corresponding to very low and high concentrations, respectively. Scale bars, 2 μm. (B) Line scans taken through the cells as illustrated by dashed white lines in (A). Calibration bar (right) is the same as in (A). Note that increases in [Ca^2+^]_i_ are localized and large in control cells, whereas they are more spread out and smaller after palmitate treatment. (C) CV of the Oregon Green 6F fluorescence ([Ca^2+^]_i_) during a 50 ms depolarization to 0 mV (0), and at rest (−70) for control cells (black, n = 6), cells treated with palmitate for 72 hr (red, n = 8), and cells treated with palmitate for 2 hr after a 72 hr culture period under control conditions (blue; n = 6). All error bars are ±SEM, ^∗^p < 0.05.

Exocytosis of insulin granules depends on localized increases in [Ca^2+^]_i_ close to the voltage-gated Ca^2+^-channels (Barg et al., 2001). We examined the effects of palmitate exposure on β cell Ca^2+^ channel distribution by measurements of near-plasma membrane [Ca^2+^]_i_ transients elicited by 50 ms depolarizations from −70 to 0 mV using the low-affinity Ca^2+^ indicator Oregon Green 6F (Figure 2A). This strategy was chosen as the Ca^2+^ channel density in mouse β cells is too low to allow immunocytochemical studies of Ca^2+^ channel distribution. Line scans obtained along the white lines are shown for one control cell and one palmitate-treated cell (Figure 2B). Whereas membrane depolarization produced discrete regions with high [Ca^2+^]_i_ in control cells, the increase in [Ca^2+^] was more diffuse in palmitate-treated cells and did not attain as high concentrations. To quantify this, we calculated the coefficient of variation (CV) of the Ca^2+^ signal in the entire cell footprint; a greater spatial heterogeneity will produce a higher CV. This analysis revealed that depolarization-evoked [Ca^2+^]_i_ (labeled “0”) is significantly less compartmentalized in palmitate-treated β cells than in control cells (Figure 2C). However, resting [Ca^2+^]_i_ prior to depolarization (labeled “−70”) was not different between control and palmitate-treated cells. The latter finding argues that the observed effect on depolarization-evoked Ca^2+^ influx cannot be attributed to differences in plasma membrane adherence to the coverslip or dye infusion. We ascertained that the amount of Ca^2+^-sensitive dye infused into the cell did not differ between the two conditions (data not shown; p = 0.35). The redistribution of [Ca^2+^]_i_ was only observed in response to long-term palmitate exposure and in β cells that were first cultured under control conditions for 72 hr and then exposed to palmitate for 2 hr, the measured CV remained the same as in control cells both at −70 and 0 mV.

The amplitude of the whole-cell Ca^2+^ current is not affected by exposure to palmitate (Olofsson et al., 2007). Likewise, there is no change in the relative contribution of L-type Ca^2+^ channels; the L-type Ca^2+^ channel blocker isradipine (10 μM) reduced the whole-cell Ca^2+^ current by 55% ± 13% (n = 4) and 57% ± 10% (n = 4) in control and palmitate-treated cells, respectively. Thus, the observed changes in Ca^2+^ entry are likely to reflect redistribution of Ca^2+^ channels rather than altered subtype expression/magnitude of the Ca^2+^ current. This conclusion is in agreement with the qPCR data (Table S1).

In separate experiments, we analyzed the distribution of syntaxin-1a in control and palmitate-treated cells; no difference was observed (CV = 0.17 ± 0.01 and 0.16 ± 0.01 in control and palmitate-treated cells, respectively; Figure S2). Palmitate was likewise without effect on the membrane distribution of SNAP-25 (data not shown).

### Inhibition of Rapid Exocytosis in Palmitate-Treated Cells

**Figure 3:**
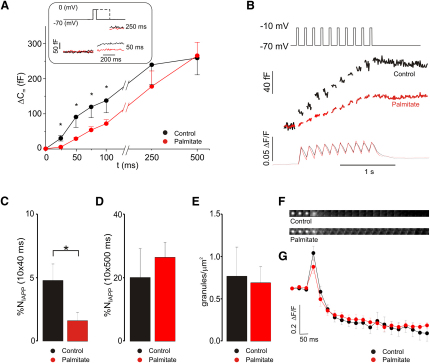
Chronic Palmitate Treatment Alters Exocytosis as Measured by Optical and Electrophysiological Methods in Single β Cells (A) Exocytosis (monitored as changes in membrane capacitance, ΔC_m_) plotted against pulse duration (t) for both control (black, n = 7) and palmitate-treated (red, n = 9; ^∗^p < 0.05) cells. The inset compares exocytotic responses elicited by a 50 or 250 ms depolarization in control (black) and palmitate-treated (red) cells. (B) Exocytosis (ΔC_m_; middle) and normalized submembrane [Ca^2+^]_i_ signals (ΔF/F; bottom) evoked in control cells (black) and palmitate-treated cells (red) by a train of ten 40 ms depolarizations from −70 to −10 mV (6.67 Hz; top). (C and D) Percentage of granules (%N_IAPP_) visible in the cell footprint released during trains of ten 40 ms (same as in B) or ten 500 ms depolarizations (1 Hz; D) for control (black, n = 7) and palmitate-treated (red, n = 8) cells (^∗^p < 0.05). (E) Granule density measured in control (black) and palmitate-treated (red) cells prior to stimulation. Same cells as in (B)–(D). (F) Single fusion events from control (top) and palmitate-treated (lower) cells recorded at 20 Hz. Each frame is 1 μm. (G) ΔF/F IAPP-mCherry signal in control (black) and palmitate-treated (red) cells. Both traces are time-aligned averages of 15 fusion events recorded in three control and three palmitate-treated cells. Images and data points in (F) and (G) have been aligned. All error bars are ±SEM.

What are the effects of Ca^2+^ channel redistribution on exocytosis? Figure 3A shows increases in membrane capacitance (ΔC_m_) measurements from control and palmitate-treated cells when exocytosis was elicited by progressively longer (25–500 ms) depolarizations from −70 to 0 mV. The inset compares the responses in control cells (black) and palmitate-treated cells (red) during 50 and 250 ms depolarizations. Whereas a substantial (>50%–80%) decrease was observed in palmitate-treated cells during short (<100 ms) depolarizations, no significant inhibition was detected during depolarizations ≥250 ms.

We next optically measured exocytosis of individual granules in parallel with measurements of submembrane [Ca^2+^]_i_ and ΔC_m_ in β cells expressing a fluorescently tagged variant of the granule protein IAPP (IAPP-mCherry; Figure S3). Exocytosis was elicited using a pulse protocol simulating glucose-induced β cell electrical activity that consisted of ten 40 ms depolarizations from −70 to −10 mV. The total ΔC_m_ evoked by the train was reduced by 55% (Figure 3B), from 118 ± 39 fF (n = 7) under control conditions to 48 ± 18 fF (n = 8; p < 0.05) after palmitate exposure. This inhibition of exocytosis occurred without any changes in overall [Ca^2+^]_i_ (compare red and black traces in the lower panel of Figure 3B (p > 0.5). Inhibition of exocytosis was also detected in the optical measurements. In control cells, 5% of the proximal (near-membrane) granules underwent exocytosis and released their fluorescent cargo during the train as compared to 1.5% in cells cultured in the presence of palmitate (Figure 3C). By contrast, no palmitate-induced inhibition of exocytosis was detectable when stimulation was elicited by depolarization of a supraphysiological duration (ten 500 ms depolarizations to zero mV; Figure 3D). Under these conditions, the total ΔC_m_ averaged 425 ± 61 fF (n = 7) and 432 ± 82 fF (n = 8) in control cells and palmitate-treated cells, respectively. In the fluorescent imaging experiments, 20%–25% of the docked granules were released during the train in both control and palmitate-treated cells (Figure 3D). The suppression of exocytosis by action potential-like stimulation in palmitate-treated cells cannot be accounted for by a dearth of available granules, which averaged ∼0.7 granules/μm^2^ in both control and palmitate-treated cells (Figure 3E). The latter value is in excellent agreement with that reported by others in human β cells (Michael et al., 2007) but different from that reported previously in rat β cells (Tsuboi et al., 2006).

Figure 3F compares the exit of the IAPP-mCherry cargo in both control (top) and palmitate-treated cells (bottom). In both cases, granule emptying is complete within four to five frames (<250 ms). Figure 3G depicts the average time courses of granule emptying in control (black) and palmitate-treated cells. The initial increase reflects the discharge of fluorescent peptide cargo into the space between the coverslip and the cell membrane, which then rapidly decays to background values as the fluorescent proteins diffuse away from the cell. Thus, FFA pretreatment does not, contrary to what was previously hypothesized (Olofsson et al., 2007), inhibit insulin secretion by interfering with the emptying of the granules.

### Effects of Palmitate Exposure in Relation to Pools of Granules

**Figure 4:**
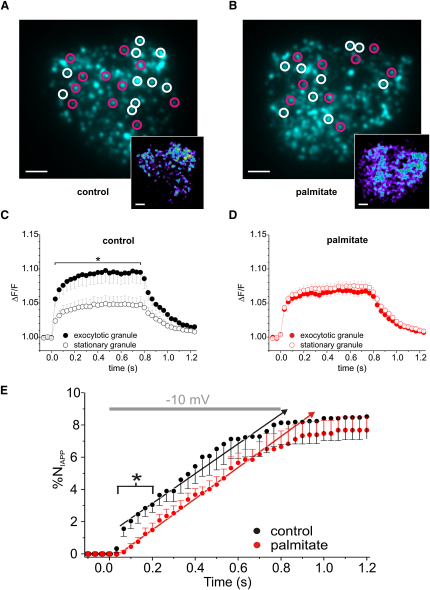
Decoupling of Ca^2+^ Entry Sites from Docked Secretory Granules Selectively Impairs Fast Exocytosis (A) Image of a single control β cell expressing IAPP-mCherry. The white and purple circles indicate granules that did or did not undergo exocytosis, respectively. The inset (lower right corner) shows the increase in [Ca^2+^]_i_ (ΔF) 33 ms after the onset of the 800 ms depolarization from −70 to −10 mV after subtraction of the resting Ca^2+^ signal in the same cell. Scale bars, 2.5 μm. (B) Same as in (A), but using a palmitate-treated cell. (C and D) Ca^2+^ increases (normalized to resting; ΔF/F) within the white (exocytosing granules) and purple (nonexocytosing granules) circles. Data are averages of 98 events in 10 control cells and 81 events in 10 palmitate-treated cells, respectively (^∗^p *<* 0.05). (E) Percentage of docked granules (N_IAPP_) in control (black) and palmitate-treated (red) cells that have undergone exocytosis displayed against time after onset of the depolarization (p < 0.05). Data were obtained from the same cells as in (C) and (D). Dotted lines indicate the rate of secretion measured >50 ms after the onset of the depolarizations. The slope of the lines is the same in control and palmitate-treated cells (p > 0.60). All error bars are ±SEM.

Granules within secretory cells belong to different functional pools (Becherer and Rettig, 2006). In addition to a RRP of granules situated close to the Ca^2+^ channels, β cells possess a slowly releasable pool (SRP) of granules that are molecularly similar to the RRP but geographically separated from sites of Ca^2+^ entry (Barg et al., 2001; Pertusa et al., 1999). To test the hypothesis that chronic palmitate exposure interferes with the coupling between the Ca^2+^ channels and granules undergoing rapid exocytosis, we simultaneously measured the rate of exocytosis and changes in submembrane [Ca^2+^]_i_ during an 800 ms depolarization (see Movie S1). Circular regions with a diameter of 1 μm were centered on each granule that underwent exocytosis during this period (white circles, Figures 4A and 4B). These regions were then used to measure the local increases in [Ca^2+^]_i_ for each exocytosing granule. The [Ca^2+^]_i_ changes (insets) in these regions were compared to that of adjacent stationary granules (purple circles). In control cells, granules that underwent exocytosis had an ∼45% larger increase in local [Ca^2+^]_i_ than those that were not released (p < 0.05, Figure 4C). No such difference existed for palmitate-treated cells (Figure 4D). We analyzed the consequence of this on the kinetics of exocytosis (Figure 4E). In control cells, 2% of the proximal granules discharged their peptide cargo during the first 100 ms of the depolarization, compared to <0.3% in palmitate-treated cells (an inhibition of >85%). At later times, however, exocytosis proceeded in parallel in control and palmitate-treated cells.

### Increased Sensitivity to Intracellular Ca^2+^ Buffering in Palmitate-Treated Cells

**Figure 5:**
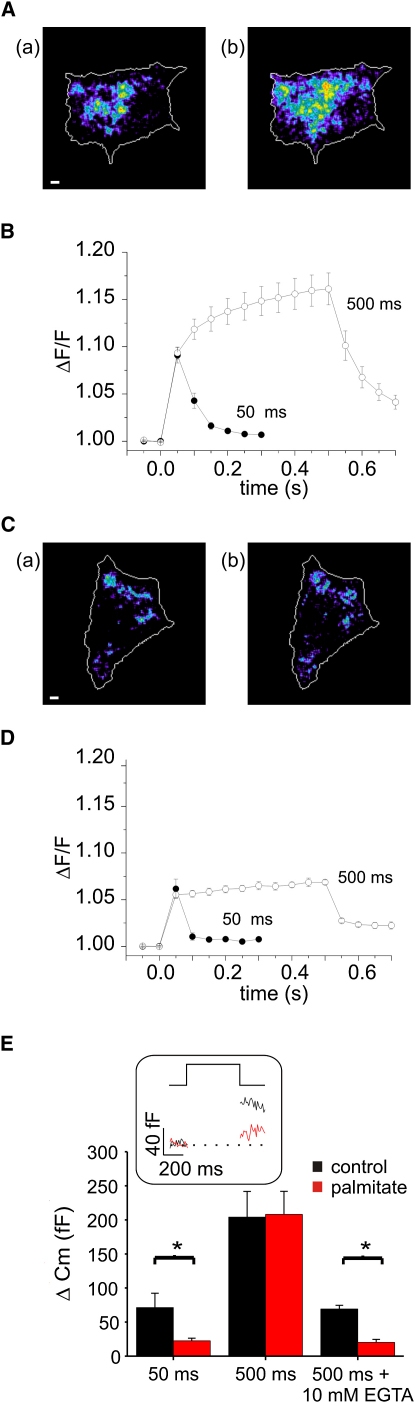
Comparison of Normalized Ca^2+^ Increases during Short and Long Stimulation under Varying Conditions of EGTA Buffering (A) Evanescent field illumination of a voltage-clamped β cell infused with EGTA (50 μM) stimulated by first a single 50 ms (Aa) and subsequently a 500 ms (Ab) depolarization from −70 to 0 mV. Changes in [Ca^2+^]_i_ are displayed in pseudocolors with black/blue and yellow/red corresponding to very low and high concentrations, respectively. Scale bars, 1 μm. (B) Mean normalized [Ca^2+^]_i_ increases (n = 4 cells). (C and D) Same conditions as in (A) and (B) except that 10 mM EGTA was included in intracellular medium (n = 4). (A)–(D) were performed on control cells. (E) Comparison of ΔC_m_ increases evoked by single 50 ms or 500 ms depolarizations to 0 mV in the presence of low intracellular EGTA (50 μM EGTA) as shown in Figure 3A were compared to responses elicited by a 500 ms depolarization in the presence of 10 mM at low (50 μM EGTA) or high (10 mM EGTA) Ca^2+^ buffering for both control (black; n = 9) and palmitate-treated (red, n = 10) cells. Inset shows exocytosis elicited by 500 ms depolarization (0 mV) in the presence of 10 mM intracellular EGTA in control (black) and palmitate-treated (red) cells. ^∗^p < 0.05. All error bars are ±SEM.

The data of Figure 4E support the notion that control cells contain both RRP (close to Ca^2+^ channels) and SRP (further away from the Ca^2+^ channels) granules, whereas palmitate-treated cells contain only SRP granules. Exocytosis of these two pools of granules should be differentially influenced by Ca^2+^ buffering; exocytosis of granules situated far away from the Ca^2+^ channels should be inhibited by the slow Ca^2+^ buffer EGTA, whereas granules residing in close proximity to the Ca^2+^ channels should be less affected (Marty and Neher, 1985). Figure 5A compares [Ca^2+^]_i_ increases elicited by a 50 (Figure 5Aa) and a 500 ms (Figure 5Ab) depolarization (0 mV) in a β cell dialyzed with 50 μM EGTA. Under these conditions, a longer depolarization expands [Ca^2+^]_i_ microdomains. Thus, there is a progressive increase in submembrane [Ca^2+^]_i_ (measured as ΔF/F) beyond that attained during the first 50 ms that reflects the intracellular diffusion of Ca^2+^. By contrast, the microdomains did not expand with pulse duration when the cells were infused with 10 mM EGTA (Figures 5Ca and 5Cb). Consequently, the increase in ΔF/F was maximal at 50 ms (Figure 5D).

In cells containing 50 μM EGTA, exocytosis evoked by 500 ms depolarizations was unaffected by palmitate, whereas the response to the 50 ms depolarization was reduced by 68% after palmitate treatment (Figure 5E; same data as in Figure 3A). When intracellular Ca^2+^ diffusion was restricted by including 10 mM EGTA in the intracellular solution, the amplitude of the response elicited by 500 ms depolarizations was reduced and became comparable to that evoked by a 50 ms depolarization at low intracellular buffering. Under these conditions, exocytosis elicited by the 500 ms depolarization was inhibited by 70% in palmitate-treated cells (inset). We conclude that the time- and diffusion-dependent expansion of the [Ca^2+^]_i_ domain explains why palmitate does not inhibit exocytosis elicited by longer depolarizations at low intracellular Ca^2+^ buffering.

### Differential Effects of Palmitate on Exocytosis Evoked by Localized and Global Elevations of [Ca^2+^]_i_

**Figure 6:**
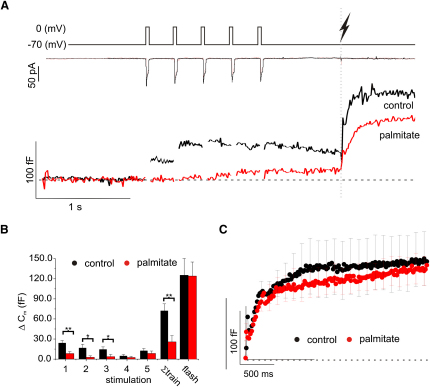
FFA Exposure Differentially Affects Exocytosis Elicited by Voltage-Clamp Depolarizations and Photoliberation of Caged Ca^2+^ (A) Representative recordings of membrane capacitance (ΔC_m_) in control (black) and palmitate-treated (red) cells (bottom) and in response to a train of five 40 ms depolarizations from −70 to 0 mV (V_m_, top) followed by photoliberation of caged Ca^2+^ by a flash of UV light (lightning) in a control cell and a palmitate-treated cell. Measurements of membrane currents (middle) are also shown. (B) Average increases in membrane capacitance (ΔC_m_) in response to each 40 ms depolarization to zero mV, the total increase evoked by the train (Σtrain) and the magnitude of the rapid component of capacitance increase produced by photoliberation of caged Ca^2+^ (flash) in control cells (black, n = 11) and palmitate-treated cells (red, n = 11; ^∗^p < 0.05 and ^∗∗^p < 0.01). (C) Average capacitance changes elicited during the first 500 ms by photoliberation of caged Ca^2+^ in control cells (black; n = 11) and palmitate-treated cells (red; n = 11). For clarity, standard errors are only shown for every twentieth data point. The horizontal line indicates the prestimulatory baseline.

We tested this idea by comparing exocytosis evoked by brief voltage-clamp pulses with that elicited by a global increase in [Ca^2+^]_i_ produced by photoliberation of “caged” Ca^2+^ from Ca^2+^—NP-EGTA in control and palmitate-treated cells (Figure 6A). Cells were first stimulated with five 40 ms depolarizations to 0 mV. In control cells, the first two to three pulses of the train evoked significant exocytosis, whereas the responses in palmitate-treated cells were almost abolished. Some recovery of exocytosis was observed in palmitate-treated cells during the fifth pulse, presumably reflecting accumulation of [Ca^2+^]_i_ within the cell (cf. Figure 3B). In both control and palmitate-treated cells, photoliberation of caged Ca^2+^ elicited robust exocytotic responses. Figure 6B summarizes the average responses for each pulse of the train, the total increase evoked by the train (Σtrain) and the response to photoreleasing caged Ca^2+^ (flash). During the first three pulses, exocytosis was inhibited by >65%, whereas the responses elicited by uncaging of [Ca^2+^]_i_ were almost identical. The increases in ΔC_m_ elicited by photorelease could be reasonably fit by double exponentials with time constants of 23 ± 5 ms and 497 ± 213 ms (n = 10) in control cells and 26 ± 4 ms and 920 ± 290 ms (n = 11) in palmitate-treated cells. The corresponding increases in ΔC_m_ for the rapid and slow components of exocytosis averaged 85 ± 5 fF and 130 ± 38 fF in control cells and 91 ± 3 and 112 ± 39 fF in palmitate-treated cells. We conclude that pretreatment with palmitate inhibits insulin exocytosis by affecting the location of Ca^2+^ influx rather than the Ca^2+^ sensitivity of exocytosis.

### Pharmacological Correction of the Inhibitory Action of Palmitate Exposure on Insulin Secretion

**Figure 7:**
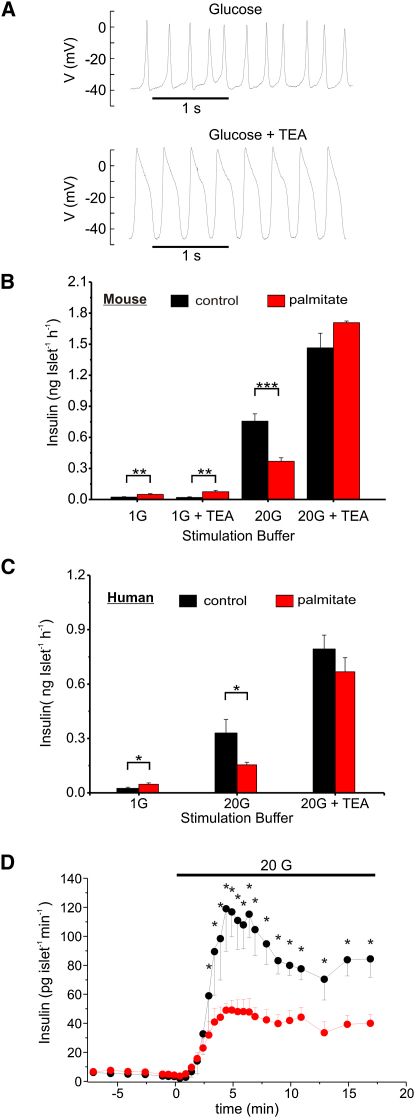
TEA Normalizes Glucose-Induced Insulin Secretion from Palmitate-Treated Islets (A) Action potentials recorded from intact islets in the presence of 20 mM glucose alone (top) and after addition of 20 mM TEA (lower). Examples shown were recorded from a palmitate-treated islet, but similar effects on action potential duration were observed in control cells. (B and C) Measurements of insulin secretion during static incubation of isolated mouse (B, n = 6 [1 mM glucose + TEA] or n = 15 [all other conditions]) or human (C, n = 6 with islets from two donors) islets with 1 or 20 mM glucose in the absence and presence of TEA as indicated during a 1 hr incubation. ^∗^p < 0.05,^∗∗^p < 0.01,^∗∗∗^p < 0.001. (D) Dynamics of glucose-induced insulin secretion in control (black) and palmitate-treated islets (red). ^∗^p < 0.05 versus control (n = 7 for both conditions). All error bars are ±SEM.

The capacitance measurements and the optical imaging of exocytosis in single β cells suggest that palmitate treatment selectively interferes with rapid exocytosis by disrupting the tight functional association between the secretory granules and the Ca^2+^ channels, whereas that elicited by longer stimuli is unaffected. If the suppression of glucose-evoked insulin secretion from intact pancreatic islets also results from this effect, then experimental maneuvers that expand the boundaries of [Ca^2+^]_i_ microdomains should rescue insulin secretion. To test this idea, we used the K^+^ channel blocker tetraethylammonium (TEA), which markedly prolongs the β cell action potential (Atwater et al., 1979). In intact islets cultured for 72 hr, the duration of the action potentials observed in the presence of 20 mM glucose alone and following addition of 20 mM TEA averaged 31 ± 1 (n = 3) and 326 ± 17 ms (n = 4), respectively (Figure 7A). In agreement with previous studies (Olofsson et al., 2007), exposure of mouse islets to palmitate for 72 hr reduced insulin secretion in response to 20 mM glucose by ∼60%. TEA (20 mM) stimulated insulin secretion in both control and palmitate-treated islets (Figure 7B). Importantly, TEA also corrected the secretion defect induced by palmitate; in the presence of the K^+^ channel blocker, insulin secretion in control and palmitate-treated islets was not different. The reduction of glucose-stimulated insulin secretion could not be attributed to a palmitate-induced decrease in insulin content, which averaged 107% ± 8% (n = 12) of its control value. Basal secretion in 1 mM glucose was marginally stimulated in palmitate-treated cells compared to control islets, and TEA had only a weak effect under these conditions. Long-term exposure to palmitate also reduced glucose-induced insulin secretion from human islets (Figure 7C) without affecting insulin content (104% ± 16% of control islets; n = 6). This inhibition was also counteracted by TEA.

We finally investigated the effects of palmitate exposure on the kinetics of glucose-induced insulin secretion from mouse islets (Figure 7D). In control islets, glucose (20 mM) produced a 30-fold stimulation of insulin secretion that peaked after 5 min and later stabilized at a somewhat lower plateau (20-fold higher than basal). In islets that had been treated with palmitate, glucose-stimulated insulin secretion was inhibited by ∼60% throughout the stimulation period.

## Discussion

Here we show that the inhibitory effect of long-term exposure to palmitate on insulin secretion is associated with alterations in the microdomains of Ca^2+^ entry in β cells. This leads to a selective inhibition of exocytosis triggered by brief (<100 ms) action potential-like depolarizations. Although the β cell contains a large number of release-competent granules, only those in close proximity to the voltage-gated Ca^2+^ channels are released during β cell electrical activity. We have previously proposed that Ca^2+^ channels are physically tethered to the insulin granules (Wiser et al., 1999). Thanks to this arrangement, a subset of release-competent granules are exposed to the very high [Ca^2+^]_i_ that occurs in the close vicinity of the Ca^2+^ channel. Our data suggest that palmitate causes disassembly of the tight complexes of Ca^2+^ channels and weakens the Ca^2+^ microdomains that form and trigger insulin exocytosis. Under control conditions, the close proximity of Ca^2+^ channels and secretory granules ensures that exocytosis is triggered by the localized increases in [Ca^2+^]_i_ evoked by an action potential (Figure S4A). Following palmitate treatment, however, the increase in [Ca^2+^]_i_ does not reach sufficiently high concentrations close to the secretory granule to produce exocytosis. This would explain the strong inhibition of exocytosis during action potential-like stimulation as well as the much greater inhibition of exocytosis caused by strong Ca^2+^ buffering (Figure 5E). This scenario also accounts for the failure of tolbutamide to correct the secretion defect (Olofsson et al., 2007). This is because tolbutamide triggers brief action potentials of the same type as those occurring in response to glucose (Henquin and Meissner, 1982).

We emphasize that insulin secretion from palmitate-treated β cells is not inhibited as a consequence of reduced release competence (Figure 6C). The fact that exocytosis during 250 ms depolarizations is unaffected is also consistent with the idea that FFAs suppress glucose-induced insulin secretion by disruption of the tight coupling between Ca^2+^ channels and the secretory granules. During such long depolarizations, [Ca^2+^]_i_ equilibrates within the cell and exocytosis will no longer be restricted to the immediate vicinity of the Ca^2+^ channels. This accounts for the ability of TEA, which leads to a marked prolongation of the β cell action potential, to restore insulin secretion in palmitate-treated islets to control values.

It may seem surprising, given that rapid exocytosis is selectively affected in the capacitance measurements and optical imaging experiments, that first-phase and second-phase insulin secretion are similarly affected (Figure 7D). Accumulating evidence suggests that first-phase insulin secretion reflects exocytosis of granules residing in close proximity to the voltage-gated L-type Ca^2+^ channels (RRP) (Rorsman and Renstrom, 2003; but see Pedersen and Sherman, 2009; Shibasaki et al., 2007). Once these granules from the RRP have undergone exocytosis, they must be replenished by physical mobilization of new granules to the Ca^2+^ channels and/or lateral movement of the Ca^2+^ channels to docked granules (Figure S4B). Importantly, both first- and second-phase insulin secretion are triggered by the Ca^2+^ influx during the brief action potentials, and they occur at a frequency too low to allow major summation of the individual [Ca^2+^]_i_ transients (Figure 3B). First-phase insulin secretion is normally larger than the second phase because the rate at which RRP is replenished is lower than the maximum secretory capacity.

We emphasize that our data do not exclude the possibility that other adverse effects (including increased cell death [Eizirik et al., 2008] and mitochondrial uncoupling [Joseph et al., 2004]) ultimately develop during protracted hyperlipidemia and contribute to the impaired insulin release. It is pertinent, however, that the mechanism we describe here, that results in reduced Ca^2+^-dependent exocytosis, develops within 72 hr and occurs before the other functional abnormalities are detectable.

What causes the Ca^2+^ channels to dissociate from the secretory granules? Our data indicate that this is not a consequence of altered membrane fluidity. Previous work (Barg et al., 2001) suggests that the tethering of the secretory granules to the Ca^2+^ channels involves the interaction of the synprint peptide (the cytoplasmic domain connecting the second and third homologous domain of the L-type Ca^2+^ channel) with the exocytotic core complex consisting of syntaxin-1, synaptotagmin, and SNAP-25. In mouse insulinoma MIN6 cells, long-term exposure to palmitate alters the expression of several proteins involved in β cell exocytosis (Lovis et al., 2008). Mice lacking syntaxin-1a exhibit a reduced number of docked granules and loss of first-phase insulin secretion but normal second-phase release (Ohara-Imaizumi et al., 2007), but our own data did not reveal any abnormalities in the expression or distribution of syntaxin-1 (Figure S3) or SNAP-25 (Zraika et al., 2004). There were likewise no major changes in the transcription of other exocytotic proteins/Ca^2+^ channel subunits that would explain the functional data (Table S1). Clearly, mRNA levels need not equate to protein levels. Chronic exposure to palmitate results in increased expression of the microRNA miR34a (Lovis et al., 2008). MicroRNAs act by inhibition of translation and may consequently affect protein expression even in the absence of any changes in gene transcription. It should be noted, however, that long-term exposure to palmitate has no effect on rapid exocytosis elicited by photorelease of caged Ca^2+^ (Figure 6). This makes it less likely that the suppression of insulin secretion reflects reduced expression of key exocytotic proteins. Alternatively, chemical modification of these proteins, via changes in acylation (Yajima et al., 2000) or palmitoylation (Fukata et al., 2006) resulting from the altered phospoholipid composition of the plasma membrane (Figure 1), may interfere with the stability of the granule/Ca^2+^ channel complexes. This will be challenging to examine, as the pharmacological inhibitors of acylation have numerous side effects including inhibition of glucose metabolism (Straub and Sharp, 2007).

What is the physiological rationale for the ability of lipids to interfere with the association of secretory granules and Ca^2+^ channels in β cells? Importantly, short-term (2 hr) exposure to palmitate (which stimulates insulin secretion; Olofsson et al., 2004) did not affect Ca^2+^ channel clustering (Figure 2C). Thus, the effect is only observed during protracted exposure. Long-term elevation of plasma FFAs occurs physiologically during fasting, when the plasma FFA concentration may increase to ∼1.5 mM (review, Frayn, 2003). In vivo recordings of β cell electrical activity have shown that the increased plasma FFA associated with fasting leads to stimulation of β cell action potential firing but suppression of insulin secretion (Fernandez and Valdeolmillos, 1998). Thus, the normal correlation between β cell electrical activity and insulin secretion is lost when plasma FFA levels are chronically elevated. Our data suggest that this results from a lipid-induced dissociation of Ca^2+^ channels from the secretory granules. It is tempting to speculate that this represents an evolutionarily conserved catabolic mechanism that suppresses insulin secretion. If it were not for this mechanism, elevation of FFAs would stimulate insulin secretion at plasma glucose levels found during protracted fuel withdrawal: 3.5 mM in man (Frayn, 2003) and 4.5 mM in mice (Fernandez and Valdeolmillos, 1998). However, this mechanism may backfire, at least in genetically predisposed individuals (Kashyap et al., 2003), when plasma lipids become chronically elevated as a consequence of a high-fat diet or impaired insulin secretion. Once this has occurred, it is easy to envisage a vicious cycle resulting in progressive deterioration of insulin secretion and elevation of plasma FFAs culminating in glucose intolerance and overt diabetes.

## Experimental Procedures

### Tissues and Cell Isolation, Electrophysiology, Hormone Release Measurements, Adenoviral Infection, and Composition of Media

All animal experiments were carried out on standard laboratory CD-1 mice. Human islets were obtained from the Oxford Islet Isolation Centre. Previously described methods were used for preparation of islets and single β cells, electrophysiology, and insulin release measurements (Olofsson et al., 2007). Palmitate was prepared in a solution bound to fatty acid-free bovine serum albumin (BSA). Palmitate was dissolved in 95% ethanol, and stoichiometric amounts of NaOH were added. The solution was dried using nitrogen gas; water was subsequently added and the solution heated to create a hot soap. The solution was stirred and BSA was added to a final concentration of 10% w/v, to create a 10x stock solution. The pH was set to 7.4 (NaOH). Islets were cultured in RPMI 1640 together with 0.5 mM palmitate in the presence of 1% BSA for 72 hr. The resulting free FFA concentration was estimated to be 26 nM. Control islets were cultured in the presence of 1% BSA alone.

Islet perifusions were performed using an in-house-designed system with four temperature-controlled chambers. Fifty size-matched islets were transferred onto a 1 μm nylon mesh cut to the size of the chamber, which was filled and perfused continuously with extracellular medium containing (in mM) 120 NaCl, 4.7 KCl, 25 NaHCO_3_, 1.2 KH_2_PO_4_, 1.2 MgSO_4_, 10 HEPES (pH 7.4 with NaOH), 2.5 CaCl_2_, 0.5 mg/ml BSA, and D-glucose as indicated. The chamber temperature was kept at 37°C. Samples were collected in 96-deep-well format plates every 30 s and kept at −20°C pending insulin measurements.

Expression of IAPP-mCherry was achieved by adenoviral infection using an in-house vector modified to express the mCherry fluorophore. Homologous recombination of the vector into the adenovirus was performed by Vector BioLabs (Philadelphia, USA).

The standard extracellular medium for imaging and electrophysiological measurements consisted of (in mM) 138 NaCl, 5.6 KCl, 1.2 MgCl_2_, 2.6 CaCl_2_, 5 HEPES, and 5 D-glucose at pH 7.4 (adjusted with NaOH). The intracellular solution consisted of (in mM) 125 Cs-glutamate, 10 CsCl, 10 NaCl, 1 MgCl_2_, 0.05 EGTA, 3 Mg-ATP, 0.1 cAMP, 5 HEPES (pH 7.2 using CsOH), and 0.03 Oregon Green 6F. In Figures 2, 5D, and 5E, intracellular Ca^2+^ buffering was increased by adding 10 mM EGTA (with iso-osmolar reduction of 10 Cs-glutamate).

Photorelease of “caged” Ca^2+^ was initiated using a JML-C2 flashlamp (Rapp Optoelektronik GmbH, Hamburg, Germany). The pipette solution consisted of (mM) 110 K-glutamate, 10 KCl, 20 NaCl, 1 MgCl_2_, 25 HEPES (pH 7.1 with KOH), 3 MgATP, 3 NP-EGTA (Synaptic Systems, Goettingen, Germany), and 2 CaCl_2_. The low-affinity Ca^2+^ indicator Oregon Green 6F was included in this medium at a concentration of 0.03 mM.

All solutions were osmotically balanced to between 290 and 310 mOsm. Experiments were performed at +32°C (electrophysiology) or +37°C (hormone release).

### Imaging of Exocytosis and [Ca^2+^]_i_

Images were obtained with a customized Olympus IX-81 total internal reflection fluorescence (TIRF) microscope equipped with a 150×/1.45 Apo lens (Olympus, UK). The 488 nm line of an argon ion laser (Melles Griot, Germany) was used to excite the Ca^2+^ dye Oregon Green 6F (Invitrogen, Carlsbad, USA), and a 561 nm line of a steady-state diode laser was used to excite IAPP-mCherry. Two excitation ports with separate focal pathways were used to make independent adjustments of each laser. The emission pathway for both fluorescent reporters was imaged simultaneously with an image splitter (Dual View, Optical Insights, Santa Fe, NM, USA) creating side-by-side images of both emission wavelengths on the chip of a charge-coupled device (CCD) camera (Cascade II 512B, Roper Scientific, Trenton, NJ, USA). All images were acquired using CellˆR software (Olympus, UK) and later analyzed with MetaMorph software version 7.53 (Universal Imaging, Downingtown, PA, USA). Images were acquired at 20–30 Hz with 10 ms excitation exposure. To correct for alignment between the two channels, live images were adjusted by hand to align the emission pathway to overlap in both channels by imaging 200 nm tetraspeck beads (Molecular Probes, Eugene, OR). This correction was done on a daily basis.

### Fatty Acid Analysis and Isotopic Enrichment

Total phospholipids and triglycerides were separated by spotting lipid extracts onto silica gel 60 (Merck) thin-layer chromatography plates using hexane:diethyl ether:acetic acid (85:15:1 vol/vol/vol) as the solvent (Folch et al., 1956). Lipid bands were visualized under UV light after spraying with 0.1% ANS (8-anilino-1-naphthalene sulphonic acid). Identification was verified using commercial standards. Total phospholipid and triglyceride bands were transferred into glass tubes and methylated at 80°C with 1.5% (triglycerides, 2 hr) or 6% H_2_SO_4_ (phospholipids, 12 hr) in methanol. Total phospholipid and triglyceride fatty acids were eluted into hexane. Separation and quantification of the fatty acid methyl esters (FAMEs) from islet total phospholipids and triglyceride were achieved using gas chromatography (Agilent 6890 GC, Agilent Technologies, UK) fitted with a 30 m × 0.53 mm (film thickness 1 μm) capillary column (RTX-Wax). Individual fatty acid peaks were identified by reference with known FAMEs. Fatty acid compositions (μmol/100 μmol total fatty acids; mol%) in these peaks were then determined.

[U-^13^C] enrichment was determined by GC-mass spectrometry (GC-MS) using a 5890 GC coupled to a 5973N MSD (Agilent Technologies, UK). The GC was equipped with a 30 m × 0.25 mm (film thickness 0.25 μm) capillary column (rTX-5) and ions with mass-to-charge ratios (m/z) of 270 (palmitate; M+0) and 286 (i.e., palmitate uniformly labeled with ^13^C; M+16) were determined by selected ion monitoring. Dwell time was 100 ms. Tracer-tracee ratios (TTRs) for [U-^13^C]palmitate (M+16)/(M+0) were multiplied by the corresponding PL or TG fatty acid concentrations to give islet tracer concentrations.

### Data Analysis

Measurements of the rate of exocytosis (Figure 4E) as well as the magnitude of release (Figures 3C and 3D) were compared between cells by normalizing to the percentage of released granules ([number released]/[number visible]). Granules were identified by an automatic Metamorph routine that identified all “spots” between 400 and 900 nm in diameter with brightness >15% above the local background of surrounding pixels. For the total granule count, large clusters of bright granules were ignored: these accounted for ∼8% of all granules in both control and palmitate-treated cells.

For measurements of Ca^2+^ influx (Figure 2), the standard deviation (σ) and mean fluorescence (μ) were measured for the entire cell footprint, which was determined from the basal fluorescence of the Oregon Green 6F dye infusion. The CV, measured as the ratio between σ and μ, was determined by measuring the Ca^2+^ signal within the footprint during membrane depolarization after subtracting the average resting Ca^2+^ signal. The resting signal for each depolarization was calculated by averaging the four frames recorded prior to depolarizing the cell. The average background image thus generated was then subtracted from that obtained during depolarizations. Increases in [Ca^2+^]_i_ (measured as ΔF) around exocytosing granules were measured within 1 μm diameter circular regions centered on the location of all identified exocytotic events within a cell (identified by the optical increase and dispersion of IAPP-mCherry fluorescence that occurred in a separate, but aligned, optical channel). These localized increases were then compared to increases of [Ca^2+^]_i_ within adjacent, but nonoverlapping (>1.5 μm but <2 μm) regions around granules that did not undergo exocytosis. To correct for irregularities in the evanescent field and amount of membrane within a measured region, all measurements of [Ca^2+^]_i_ increases (ΔF) were normalized to basal fluorescence (F) levels prior to depolarization for each region.

Data are presented as mean values ± SEM for the indicated number of experiments (n). Statistical significance measurements were performed by Student's t test, with Bonferroni corrections in Table S1, and in Figures 1 and 7 an ANOVA test with Dunnet C post-hoc adjustments.

## Supplemental Data

Document S1. Four Figures and One Table

Movie S1. Discharge of Peptide Cargo and Elevations in Submembrane [Ca2+]_i_ within the Evanescent Field of Illumination during an 800 ms Depolarization Shown is a single β cell visualized simultaneously in two channels (Oregon-Green 6F 10 μM [left] and vesicles labeled with IAPP-mCherry [right]) during an 800 ms depolarization to −10 mV. Images were acquired at a rate of 30 Hz. Total duration of movie is 4 s.
