# Supplementary material for: Chronic Palmitate Exposure Inhibits Insulin Secretion by Dissociation of Ca2+ Channels from Secretory Granules
Source: Cell Metab. 2009 Dec 2;10(6):455–65. doi: 10.1016/j.cmet.2009.09.011 (PMC2814048; doi:10.1016/j.cmet.2009.09.011)
Supplement: Document S1. Four Figures and One Table [file mmc1.pdf]

Cell Metabolism, Volume *10*

Supplemental Data

Chronic Palmitate Exposure Inhibits Insulin Secretion by Dissociation of Ca^{2+} Channels from Secretory Granules

Michael B. Hoppa, Stephan Collins, Reshma Ramracheya, Leanne Hodson, Stefan Amisten, Quan Zhang, Paul Johnson, Frances M. Ashcroft, and Patrik Rorsman

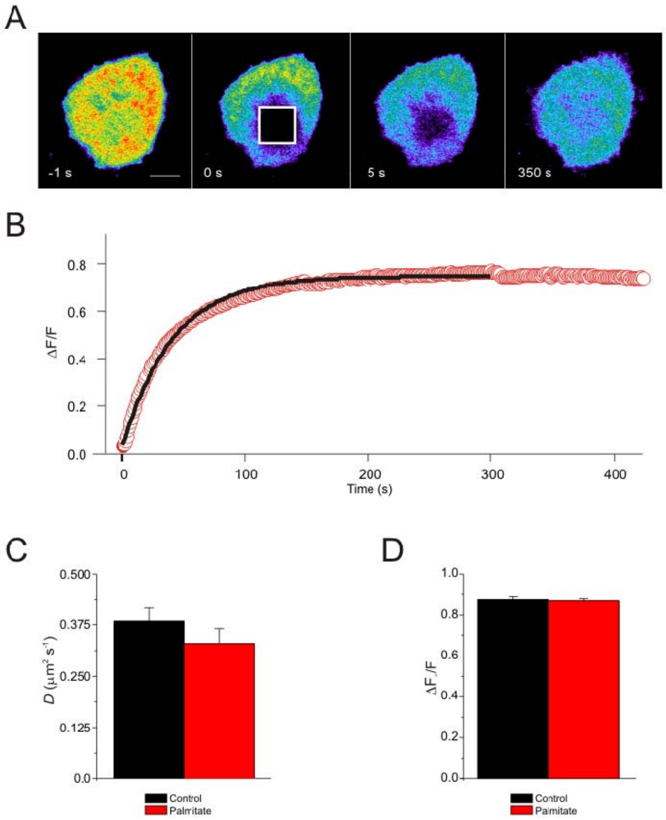

Figure S1. **No effect of palmitate treatment on membrane fluidity. A)** Confocal images of the footprint of a BODIPY-labeled β-cell before (-1 s), just after photobleaching (0) and following 5 and 350 s of fluorescence recovery. The time-

dependent recovery of ΔF/F observed reflects movement of the fluorophores into the bleached area from the surrounding unbleached region. The photobleached area is indicated by the white square (5 x 5 μm). **B)** Average fluorescence recovery time courses for a representative palmitate-treated cell. **C-D)** Diffusion coefficient (D) and mobile fraction (ΔF/F) measured in control (n=20, black) and palmitate-treated (n=20, red) cells. The values of ΔF/F (P=0.35) and D (P=0.31) were not different in control and palmitate-treated cells.

Methods: FRAP (fluorescence recovery after photobleaching) experiments were performed at room temperature utilizing the FRAP protocol of the Zeiss LSM 510 Meta Imaging system with excitation from a 488 nm argon gas laser and emitted collected through a GFP filter set. BODIPY 4,4 difluoro-5-methyl-4-bora-3a, 4a-diaza-s-inda-dodecanoic acid (Invitrogen, USA) was prepared as a 100x concentrated stock solution in DMSO. Cells were labelled with 1 μM of the fluorophore in serum-free OPTIMEM medium (Invitrogen) at 37°C for 5 min. They were then superfused for 10 min with solution containing (mM) 138 NaCl, 5.6 KCl, 2.6 $CaCl_2$, 1.2 $MgCl_2$, 5 HEPES and 5 D-glucose to remove excess BODIPY. The rate of fluorescence recovery was fit using a single exponential calculated with the Back Multiplication Method using freeware written by Kota Miura (Centre for Molecular and Cellular Imaging, EMBL Heidelberg, Germany).

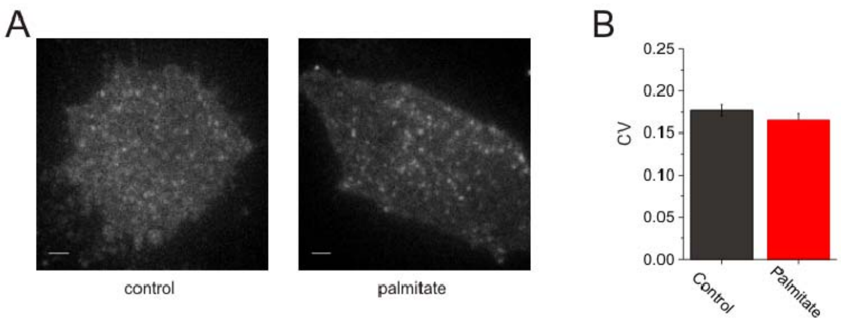

Figure S2. **A-B)** Evanescent-field illumination of β-cells cultured under control conditions (A) or in the presence of palmitate for 72 h (B) were blocked in 5% goat serum for 1 h and then immunostained with an antibody against syntaxin-1 (product # 110 011 from Synaptic Systems, Germany) for 2 h at room temperature followed by a 3x wash in PBS. Syntaxin-1 immunoreactivity was visualized using a goat anti-mouse secondary antibody (Vector Laboratories, California USA). Scale bar = 1.5 μm. **C)** Coefficient of variation for syntaxin-1 distribution in control (black; n=9) and palmitate-treated β-cells (red; n=7), $P>0.05$.

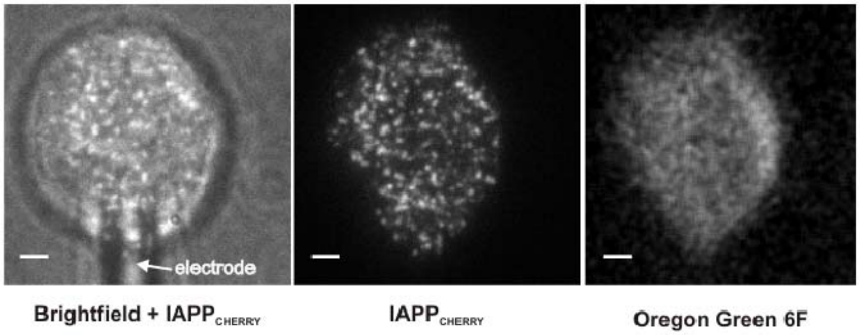

Figure S3. **TIRF imaging of Ca^{2+} and IAPP in a patch-clamped β-cell.** (Left) A voltage-clamped mouse β-cell under brightfield illumination (lthe laser was turned on to allow visualization of the tagged granules). (Middle) IAPP-mCherry labeled vesicles. (Right)) $[Ca^{2+}]_i$ measured using Oregon Green 6F. Scale bar: 2 μm.

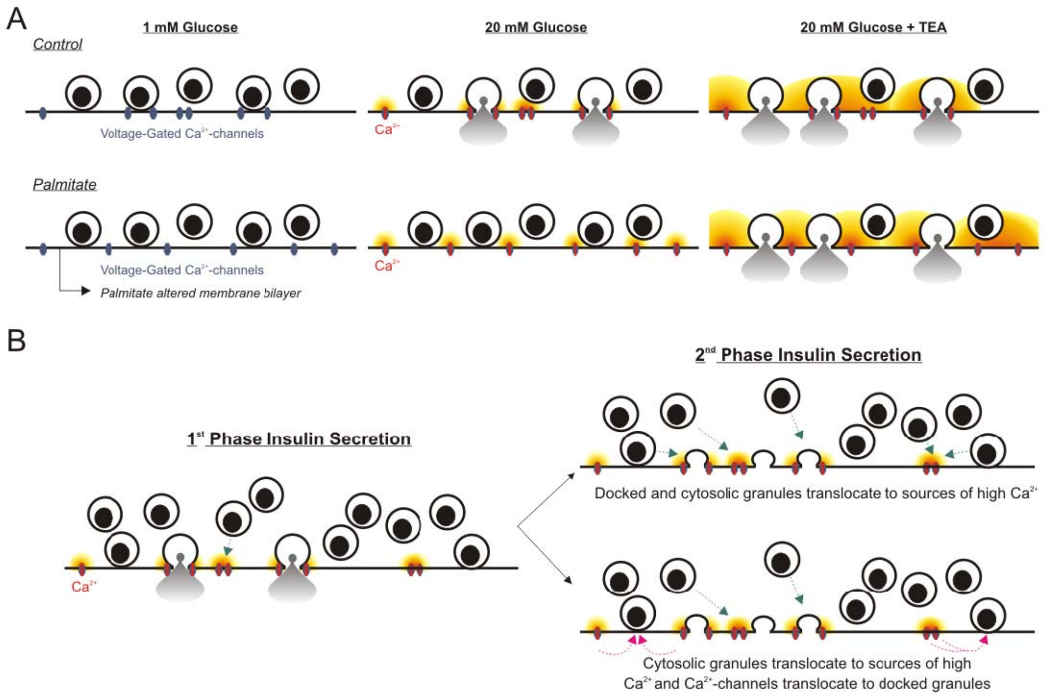

Figure S4. **Dissociation of docked granules and microdomains of Ca^{2+} influx**. **A)** Schematic of the arrangement of Ca^{2+}-channels and granules at the plasma membrane in control and palmitate-treated β-cells. Under physiological stimulation action potentials briefly open voltage-gated channels and only granules docked close to Ca^{2+} microdomains that arise during Ca^{2+}-channel opening undergo exocytosis. The impaired coupling between docked granules and Ca^{2+}-channels in palmitate treated cells impairs exocytosis. TEA-induced prolongation of the action potential expands the Ca^{2+}-microdomains and tight association of Ca^{2+}-influx and the secretory granules is no longer crucial for insulin secretion to occur. **B)** Relationship between Ca^{2+}-channels and secretory granules during 1st and 2nd phase insulin secretion. During 1st phase insulin secretion, granules situated close to the Ca^{2+}-channels (RRP) undergo exocytosis. Depletion of RRP results in the end of 1st phase secretion. During 2nd phase insulin secretion, new cytosolic granules as well as docked granules are recruited to the release

sites (top). Alternatively, not only are cytosolic granules recruited to sources of high Ca^{2+}, but also the Ca^{2+}-channels move laterally within the plasma membrane to docked granules that were previously situated too far away from the points of Ca^{2+}-entry to undergo exocytosis (bottom). Thus, both 1st and 2nd phase insulin secretion requires a tight association between the Ca^{2+}-channels and the secretory granules. 2nd phase secretion will proceed at a lower rate than 1st phase secretion because lateral movement and/or recruitment of new granules occurs at a lower rate than the maximum secretory rate.

Snap25	synaptosome-associated protein of 25,000 daltons	1.29±0.15
Syt7	synaptotagmin 7	1.57±0.32
Syt9	synaptotagmin 9	1.19±0.47
Unc13b	munc 13b	1.31±0.27
Rim2	Rim 2	1.18±0.16
Cpx1	Complexin 1	0.91±35
Sytl4	Granuphilin	0.71±0.11*
Rab3a	Rabphilin 3a	1.21±0.28
Rab27a	Rabphilin 27a	0.96±0.11
Bsn	Bassoon	1.04±0.22
Cacna1c	L-type Ca^{2+}-channel α-subunit	0.96±0.13
Cacnb2	L-type Ca^{2+}-channel β-subunit	0.93±0.08
Cacnb3	L-type Ca2+-channel β-subunit	1.17±0.17
Tcf7l2	transcription factor 7-like 2 gene	1.23±0.26

Table S1. **Expression of genes associated with exocytosis in control and palmitate-treated mouse pancreatic islets**. Gene expression data relative to GAPDH from control and palmitate-treated islets. Gene expression has been normalised to control (=100%) for nine paired experiments (each experiments based on islets from the same animal). **P*<0.05. Additional genes that were previously analyzed (Olofsson et al., 2007) and found not to change in response to palmitate exposure to have no significant correlation but not shown include Chgb, Cpt1, Ins1 and Stx1a. For all measurements, the total mouse islet RNA was purified, transcribed into cDNA and used as template for qPCR using QuantiTect primer Assays and QuantiFastqPCR enzyme (Qiagen, Venlo, the Netherlands).
